# Pregnant with metastatic neuroendocrine tumour of the ovary: what now?

**DOI:** 10.3332/ecancer.2012.240

**Published:** 2012-01-03

**Authors:** B Pistilli, CM Grana, N Fazio, A Cavaliere, ME Ferrari, L Bodei, SM Baio, G Scambia, G Paganelli, FA Peccatori

**Affiliations:** 1 Department of Medical Oncology, Ospedale di Macerata and Fertility and Procreation in Oncology Unit, Department of Medicine, European Institute of Oncology, Milan, Italy; 2 Division of Nuclear Medicine, European Institute of Oncology, Milan, Italy; 3 Upper Gastrointestinal Tumor Unit, Department of Medicine, European Institute of Oncology, Milan, Italy; 4 Department of Obstetrics and Gynecology, Catholic University, Rome, Italy; 5 Medical Physicist, Department of Medical Physics, European Institute of Oncology, Milan, Italy; 6 Division of Nuclear Medicine, European Institute of Oncology, Milan, Italy; 7 Department of Obstetrics and Gynecology, Catholic University, Rome, Italy; 8 Fertility and Procreation in Oncology Unit, Department of Medicine, European Institute of Oncology, Milan, Italy

## Abstract

Neuroendocrine tumours (NET) are a heterogeneous group of neoplasms commonly occurring in the gastrointestinal tract or lungs but can occur in other regions. Primary ovarian NET account for 5% of all NET and 0.1% of all ovarian malignancies. In metastatic disease, the therapeutic goal is to extend survival and to improve quality of life. As these tumours express somatostatin receptors, somatostatin analogues are frequently used to control symptoms. Here we present a case of a pregnant woman with an ovarian NET with liver metastases and carcinoid syndrome who was treated with the somatostatin analogue, Octreotide LAR. We also summarize reported data of the use of somatostatin analogues during pregnancy.

## Introduction and background

Neuroendocrine tumours (NET) are a heterogeneous group of neoplasms commonly located in the gastrointestinal tract or lungs. Their incidence is less than five cases/100.000 per year and it has been increasing over the last three decades [1,2]. Primary ovarian NET account for 5% of NET and 0.1% of all ovarian malignancies [3].Five years overall survival is not well established, but exceeds 70% in some series.

Neuroendocrine cells secrete various hormones (serotonin, histamine, dopamine, substance P, neurotensin, prostaglandins) which are commonly inactivated by liver monoamine oxidases. Approximately 5% of patients affected by metastatic disease experiences carcinoid syndrome: cutaneous flushing, abdominal pain, diarrhoea and bronchospasm. Classical signs and symptoms usually occur when large liver metastases are present or when these amines pass directly into systemic circulation [4]. A carcinoid syndrome has been described in around 30% of ovarian carcinoids [5]. Either short- or long-acting somatostatin analogues (SSA) (Octreotide, Octreotide LAR, lanreotide) are routinely administered for preventing carcinoid syndrome and for inhibiting tumour growth [6,7]. Moreover, in last decade treatment with radioactive-labelled somatostatin analogues [90-Y-DOTATOC and ^177^LuDOTATATE] have been developed in order to improve symptoms of hormone hypersecretion and slow tumour growth [8,9].

Although the average age of carcinoid patients is 60 years, diagnosis can occur also during reproductive age [1,4]. Due to the changes in reproductive behaviour, with increased age at first and subsequent pregnancies and to the long-term survival of patients with NET, it is not exceptional that patients ask for advice about pregnancy or that they face unexpected pregnancies during treatments.

Few data on clinical and biological effects of SSA during pregnancy have been reported so far. In most cases, the drug is withdrawn before a planned pregnancy or immediately after its confirmation [10]. However, there are a number of published reports on pregnant acromegalic women, who received SSA either throughout the whole pregnancy or during part of it [11–13].

## The clinical case

A nulliparous 31-year-old woman affected by ovarian NET with liver metastases and carcinoid syndrome was found to be 6 weeks pregnant by urine HCG test, while on Octreotide LAR 30 and 2 months after the 6th cycle of peptide receptor radionuclide therapy (PRRT) with ^177^Lu-DOTATATE ([^177^Lu-DOTA-Tyr3]-octreotate). The patients had regular menses during the previous year, and had not used contraceptive in the last 6 months.

Diagnosis of ovarian NET with liver metastases occurred 7 years earlier, when she started complaining of generalized flushing, diarrhoea and abdominal pain. In 2003, after a diagnostic laparotomy, she received six cycles of cisplatin and etoposide every 3 weeks with partial tumour regression, followed by Octreotide LAR (30 mg per month).

**Figure 1:**
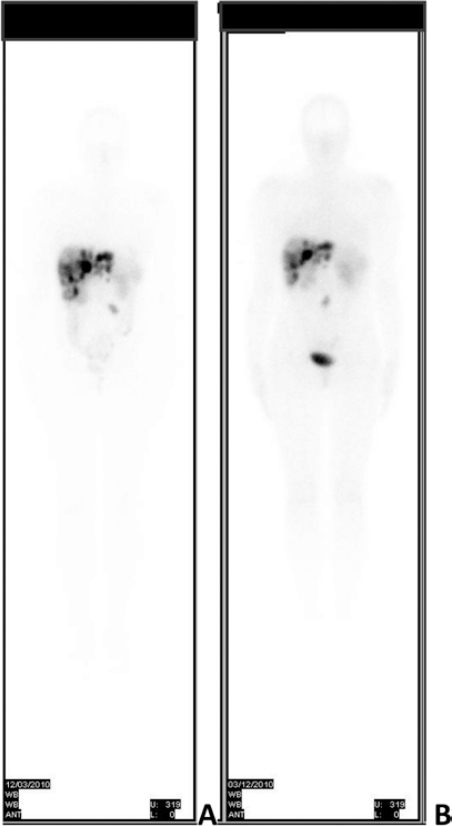
Whole-body scan with ^177^Lu-DOTATATE performed 3 months before pregnancy and after the 6th cycle of PRRT (A) and after the 8th cycle of PRRT (B).

In September 2008, after hepatic and pelvic disease progression, she underwent right ovariectomy and peritoneal node dissection. Histological examination confirmed a NET involving the right ovary, with a proliferation index of 10% (Ki-67). Subsequently, she received PRRT with ^177^Lu-DOTATATE associated with intra muscular monthly Octreotide LAR. Three months after the sixth cycle of PRRT, (cumulative activity dose 22.42 GBq – 606 mCi) (Figure 1A) an unplanned pregnancy was found. A dosimetric evaluation confirmed a very low risk of uterine irradiation (< 1 m Sv) at conception.

After onset of pregnancy, she experienced a significant worsening of symptoms, consisting of recurrent flushing, abdominal cramping, diarrhoea, severe orthostatic hypotension. No biochemical evaluation of vasoactive amines was performed. The patient was informed within a multi-disciplinary consultation about the possible maternal risks of continuing pregnancy and withdrawing SSA. The patient was also informed about the paucity of clinical data on the effects of Octreotide LAR on foetal integrity and growth. According to her desire to maintain the pregnancy, Octreotide LAR was stopped, oxatomide and ranitidine were started to limit symptoms associated with carcinoid syndrome and short-acting somatostatin analogue was suggested in case of worsening of maternal symptoms. However, spontaneous abortion occurred at 12th week of pregnancy, before any short-acting somatostatin analogue administration was attempted. Soon after miscarriage, the patient experienced a dramatic improvement in symptoms, with complete resolution of flushing.

After uterine revision, which yielded endometrial tissue without any embryonic residual, PRRT was completed with two cycles of ^177^ Lu-DOTATATE, achieving the total activity of 29.4 GBq (796 mCi) and Octreotide LAR was reintroduced with disease stabilization (Figure 1B). On her last clinical examination, 8 years after diagnosis, the patient, in good clinical condition, firmly expressed her desire to have a child in the next future, although she had concerns about the risk of symptoms worsening and flushing recurrence, as experienced in her previous gestation. Moreover, we discussed with her the new therapeutical strategies in order to further control the disease, as she has reached the maximum administrable activity of ^177^Lu-DOTATATE.

## Discussion and conclusions

Generally, NET are slow-growing malignancies. Even when metastases occur, they often exhibit an indolent clinical course, with an overall 5-year survival of 67%, across all stages and sites [1].Although the median age at diagnosis is usually in the sixth decade, they might occur also in young women, particularly when they involve the appendix or the ovary. Few anecdotal cases of NET in pregnancy have been reported in literature: most of them did not require treatment with somatostatin analogues, because of non-functioning tumours or because radical surgical resection was performed. Regarding the safety and feasibility of SSA in pregnancy, limited data are available, mainly on acromegalic patients (Table 1). Fassnacht and colleagues [11] reported the case of a young acromegalic woman who received Octreotide LAR throughout her pregnancy. RIA assessment of Octreotide in umbilical cord blood indicated a substantial materno-foetal circulation of the drug. Nevertheless, both placenta and foetus developed normally. Maffei and colleagues [13] described the case of an acromegalic woman treated with high dose of short-acting Octreotide throughout the whole pregnancy. A transient decrease in uterine artery flow after Octreotide injections was detected, without effects on pregnancy and foetal development. Finally, Boulanger *et al* reported an uneventful pregnancy in a patient affected by pancreatic beta cell hyperplasia (nesidioblastosis) treated with Octreotide during the whole gestation[14].

Despite the expression of all five somatostatin receptors (SST1, SST2, SST3, SST4 and SST5) in human placenta of acromegalic and healthy women, a very low SSA binding to placenta and umbilical cord tissues has been observed. Previous in vitro and in vivo studies demonstrated that SSA bound with low affinity to human placental receptors and the absence of changes in human placental growth hormone (hPGH) and insulin growth factor-I (IGF-I) during Octreotide treatment in pregnancy [12]. Based on these findings, we told the patient that information about safety and feasibility of SSA during pregnancy were limited and suggested short-acting SSA, because this formulation is better manageable if any pregnancy complication occurs.

However, other aspects should be taken into account in pregnant women affected by functioning neuroendocrine tumours. Pregnancy induces an altered endocrinal milieu which could influence tumour progression, particularly when an endocrine responsive tumour is present. No data about NET progression during pregnancy have been reported (14, 20), but caution is mandatory. Our patient experienced an increase in carcinoid syndrome which significantly worsened during gestation. A similar finding was described by others in patients diagnosed with acromegaly or carcinoid syndrome during pregnancy [13,15]. Consistently high levels of vasoactive peptides have been associated with late onset cardiac lesions in 50–60% of patients with carcinoid syndrome [16]. 5-HIAA (5-hydroxyindolacetic acid) and serotonin levels correlate with the risk of developing right cardiac fibrosis and mitral valve stenosis. Somatostatin analogues are the mainstay of treatment in these cases. Thus, cardiac function and potential effects of SSA withdrawn should be accurately evaluated in pregnancy, also considering the cardiac load induced by pregnancy. Recently, the placental production of serotonin was described in pregnant women. Low maternal serotonin levels might influence foetal neurogenesis, including forebrain development, and were possibly related to mental disorders in adult offsprings[17]. A possible foetal neurotoxicity cannot be ruled out when SSA are administered during pregnancy, so this should be addressed during the counselling. Finally, functioning NET might create unexpected hemodynamic changes under anaesthesia. This condition, called carcinoid crisis is a life-threatening variant of carcinoid syndrome, characterized by severe hypotension (rarely hypertension) and bronchospasm. Therefore, an adequate planning of delivery in these patients is strongly recommended [15].

In conclusion, the opportunity of future conception could be considered in women with a history of syndromic NET, but all possible risks of getting pregnant should be critically discussed in details with the patient and a multi-disciplinary support should be offered both before and during pregnancy. Reproductive issues have become more frequent even in patients with rare tumors and caregivers should be ready to face these needs even without the support of solid evidences.

## Figures and Tables

**Table 1:** Previously reported data of SSAs treatment while on pregnancy

**Authors**	**Mother disease**	**Number of pregnancies**	**Type of SSA dose**	**SSAs exposure period/total week of exposure**	**Delivery**	**Miscarriage/complications**
Cozzi R [10]	Acromegaly	2/7	Lanreotide	1st trimester/4 weeks	At term	none
Fassnacht [11]	Acromegaly	1	Octreotide LAR 10–20 mg	Whole pregnancy	38th week	Foetal growth retardation
Maffei [13]	Acromegaly	1	Octreotide 100 μg/1–2 h (1200–2400 μg/24 h)	Whole pregnancy	38th week	None
Le [15]	Carcinoid syndrome	1	Octreotide LAR and Octreotide 25 μg/h iv during delivery	Partial gestation (unknown period) and delivery	37th week	Mild foetal metabolic alkalemia
deMenis [18]	Acromegaly	1	Lanreotide 30 mg	1st trimester/4 weeks	38th week	None
Mikhail [19]	Acromegaly	1	Octreotide 100 μg t.i.d.	Whole pregnancy	38th week	None
Mozas [20]	Acromegaly	1	Octreotide 100 μg t.i.d.	1st and 2nd trimester/24 weeks	ns	None
Caron [21]	TSH-macroadenoma	1	Octreotide 300 μg die (continuous infusion)	1st and 3rd trimester/28 weeks	8th month	None
Boulanger [14]	Nesidioblastosis	1	Octreotide 1000 μg in the first trimester	Whole pregnancy	32 weeks	None
